# Primary omentum extraskeletal osteosarcoma in a dog: case report

**DOI:** 10.29374/2527-2179.bjvm000423

**Published:** 2023-10-02

**Authors:** Caroline Tessmer Elias Soares, Fernanda Peres Medeiros, Raul Martins

**Affiliations:** 1 Veterinarian, Curso de Pós-graduação Lato-sensu em Ultrassonografia de Pequenos Animais, Faculdade Método de São Paulo (FAMESP), São Paulo, SP, Brazil.; 2 Veterinarian, DSc., Coordenadora do curso de Pós-graduação Lato-sensu em Ultrassonografia Veterinária de Pequenos Animais, FAMESP, São Paulo, SP, Brazil.; 3 Veterinarian, MSc. Coordenador do curso de Pós-graduação Lato-sensu em Ultrassonografia Veterinária de Pequenos Animais, FAMESP, São Paulo, SP, Brazil.

**Keywords:** ultrasound, extraskeletal osteosarcoma, dog, ultrassonografia, osteossarcoma extraesquelético, cão

## Abstract

A rescued male mixed-breed dog, approximately nine years old, was evaluated due to progressive weight loss and an enlarged abdomen. An ultrasound revealed a large, indeterminate mass with mineral-like margins visible on the radiographs. The animal underwent an exploratory laparotomy, and the mass was excised. Histopathological analysis revealed characteristics consistent with a primary omentum extraskeletal osteosarcoma. This rare neoplasm, originating from mesenchymal cell proliferation and bone matrix production, is highly malignant. It often results in death due to metastasis and local recurrence or necessitates euthanasia post-diagnosis in certain cases.

## Resumo

Um canino macho de aproximadamente 9 anos de idade, sem raça definida, foi recebido para atendimento apresentando sinais de emagrecimento progressivo e aumento de volume abdominal. Um exame ultrassonográfico revelou uma formação de grandes dimensões e de origem indeterminada, apresentando característica de mineralização periférica pela radiografia. Foi realizada laparotomia exploratória com remoção da formação. A análise histopatológica revelou características compatíveis com osteossarcoma primário de omento, uma neoplasia rara originada da proliferação de células mesenquimais com produção de matriz óssea, com alto grau de malignidade, geralmente levando à óbito devido a metástases e recorrência local do tumor ou eutanásia após o diagnóstico em alguns casos.

## Introduction

Osteosarcoma is a highly malignant bone neoplasia, characterized by the proliferation of mesenchymal cells and the production of woven bone tissue and osteoid matrix (Leonardi, 2022). It can originate from bone, which is the most common presentation, and can be divided into axial and appendicular forms. Alternatively, it can present in an extraosseous form, which is less common and does not involve primary bone tissue (Langenbach et al., 1998; Leonardi, 2022; Thompson & Dittmer, 2017). This form primarily affects the mammary glands (Langenbach et al., 1998; Leonardi, 2022) and viscera (Patnaik, 1990). Osteosarcoma has been reported in various tissues and organs, including the mammary and salivary glands (Langenbach et al., 1998; Leonardi, 2022), liver, spleen, and urinary system (Langenbach et al., 1998; Leonardi, 2022, Patnaik, 1990), thyroid glands, skin, subcutaneous tissue, and muscle (Langenbach et al., 1998, Leonardi, 2022), gastrointestinal tract (Langenbach et al., 1998; Leonardi, 2022; MacKenzie et al., 2012; Patnaik, 1990), adrenal glands (Patnaik, 1990), eye and omentum (Langenbach et al., 1998; Patnaik, 1990), and retroperitoneal space (Piedra-Mora et al., 2016).

Extraosseous osteosarcoma is typically diagnosed in elderly patients (Kuntz et al., 1998; Langenbach et al., 1998; Thompson & Dittmer, 2017). Unlike skeletal osteosarcomas, it does not correlate with large breed dogs (Thompson & Dittmer, 2017). No breed (Leonardi, 2022; Patnaik, 1990) or sex (Langenbach et al., 1998) predilection has been observed.

Typically, its presentation involves rapid growth formation, characterized by irregular mineralized areas, as well as hemorrhagic and necrotic regions (Leonardi, 2022; Patnaik, 1990). These formations exhibit a high rate of local recurrence and metastasis (Leonardi, 2022; Thompson & Dittmer, 2017), with distant metastasis observed in up to 64% of cases (Kuntz et al., 1998; Patnaik, 1990). However, lung metastasis is relatively uncommon compared to skeletal osteosarcomas (Langenbach et al., 1998; Thompson & Dittmer, 2017). This condition generally has a poor prognosis and a short survival time post-diagnosis, possibly due to late detection (Guim et al., 2019; Thompson & Dittmer, 2017). The symptoms are typically nonspecific and directly related to the tumor’s location (Kuntz et al., 1998; Langenbach et al., 1998), and surgical access is often limited (Guim et al., 2019; Thompson & Dittmer, 2017). The prognosis is particularly poor for intra-abdominal occurrences (Langenbach et al., 1998). Death usually results from local recurrence (Langenbach et al., 1998; Thompson & Dittmer, 2017) or euthanasia following diagnosis (Piedra-Mora et al., 2016; Thompson & Dittmer, 2017).

A diagnosis is typically established through the integration of clinical, radiographic, and histological findings (Gârjoabă et al., 2009; Guim et al., 2019). This process is effectively supplemented by abdominal ultrasound, which facilitates guided biopsies or cytology (MacKenzie et al., 2012; Urbiztondo et al., 2010). It also aids in pinpointing the exact location of the tumor and any potential metastatic lesions (MacKenzie et al., 2012). From a histological perspective, certain criteria must be satisfied to affirm the diagnosis. These include a consistent morphological pattern of sarcomatous tissue, ruling out the likelihood of a mixed mesenchymal tumor; the generation of woven bone or malignant osteoid tissue; a high mitotic index; and the exclusion of bone origin (Kuntz et al., 1998; MacKenzie et al., 2012; Patnaik, 1990; Piedra-Mora et al., 2016; Urbiztondo et al., 2010).

Despite the lack of definitive information in most current literature, no specific and effective treatment has been established. When feasible, complete or partial excision of the tumor is recommended, contingent on surgical viability (Duffy et al., 2017). Outcomes and prognosis improve when complete removal is achieved (Duffy et al., 2017). Optimal results have been observed when surgery is combined with chemotherapy (Kuntz et al., 1998), although there is a need for additional studies and significant statistics to establish a definitive protocol (Duffy et al., 2017; Kuntz et al., 1998). Following diagnosis, disease staging is essential to determine the most effective treatment and prognosis for each patient (Duffy et al., 2017).

This study presents a confirmed case of primary omentum extraskeletal osteosarcoma in an elderly male dog. The diagnosis was established through ultrasonography, radiography, and histopathology. The patient was monitored from the initial diagnostic procedure through to euthanasia and necropsy. The case largely aligned with the characteristics outlined in existing literature, with the only deviation being a slightly extended post-surgical survival time than previously reported.

## Case report

**Figure 1:**
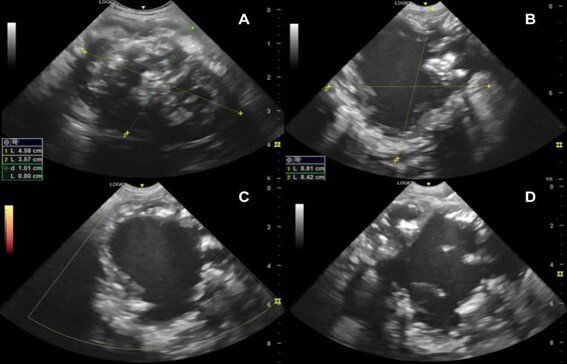
Ultrasonographic images demonstrating the characteristics of the abdominal formation in different incidences. (A) Formation with heterogeneous aspect and irregular and ill-defined margins, permeated with several hyperechogenic spots; (B) and (D) Other incidences evidencing the anechoic center and hyperechogenic and irregular margins; (C) Doppler evaluation, with no evidence of vasculature in this incidence.

A medium-sized, mixed breed male dog, approximately nine years old, was brought in for treatment following a recent rescue. The dog had a history of progressive weight loss and an enlarged abdomen. A physical examination was conducted, revealing stable vital signs: a heart rate of 120 beats per minute, 31 respiratory movements with clear respiratory sounds, and a rectal temperature of 38.2 ºC. Blood tests were performed, indicating leukocytosis with elevated monocytes and neutrophils, as well as hypoalbuminemia. No other significant changes were observed in the remaining test results. During abdominal palpation, a large mass was identified. The patient was subsequently referred for an abdominal ultrasound, which was conducted the following day. The ultrasound revealed a large, irregular formation that was heterogeneous in nature and exhibited mixed echogenicity. The formation had an anechoic center and hyperechoic spots scattered around the margins (Figure 1). The formation measured approximately 8.81 × 8.42 cm at its longest point and displayed minimal peripheral vasculature, which was difficult to delineate via Doppler evaluation. Due to the formation’s substantial size, it was impossible to determine the extent of involvement, proliferation, and/or adhesion to adjacent organs and structures.

**Figure 2:**
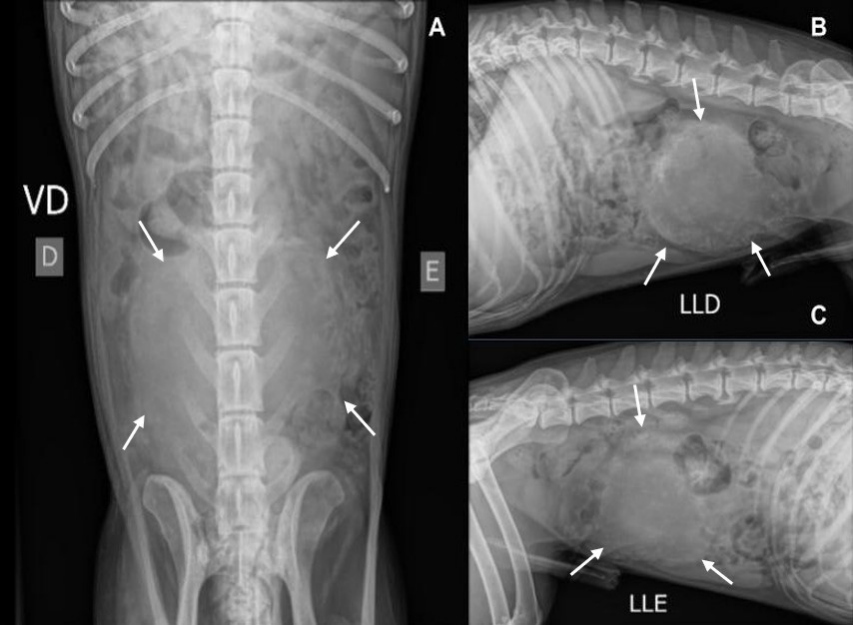
Abdominal radiographs, showing the mass. (A) Ventrodorsal (VD - ventro-dorsal) view, evidencing the homogeneous formation in the medial and caudal abdominal regions (white arrows); (B) Right lateral view (LLD - látero-lateral direita); and (C) Left lateral view (LLE - látero-lateral esquerda), both showing the formation with homogeneous center and contour with menral radiopacity spots (white arrows).

In a supplementary assessment, abdominal radiographs were conducted to identify the source of the mass. A uniform mass, characterized by peripheral mineral radiopacity spots, was detected. The primary differential diagnoses included neoplasia, granuloma, or abscess (Figure 2). Further investigation, either through computed tomography or exploratory laparotomy, was recommended.

**Figure 3:**
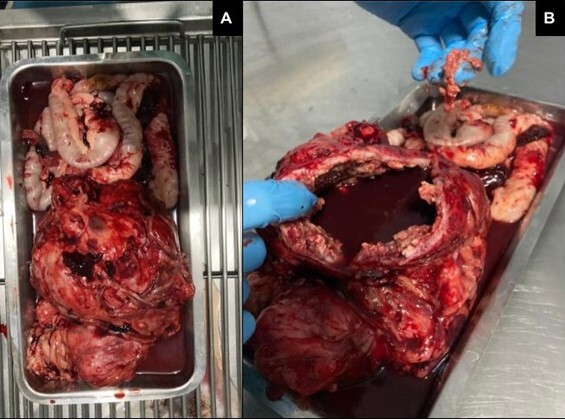
Macroscopic images of the formation, after surgical resection. (A) Structure removed during exploratory laparotomy; due to adherences, a jejunal segment was removed along with the formation; (B) Image exhibiting firm margins permeated with small mineral lesions and the center of hemorrhagic and necrotic material.

**Figure 4:**
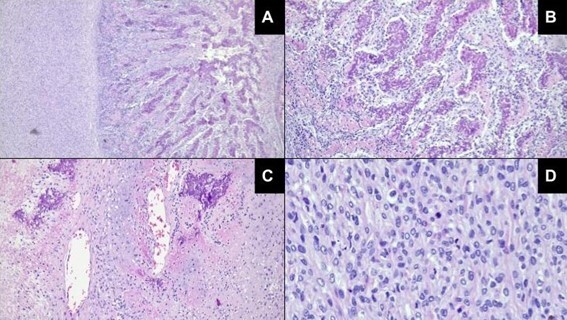
Images exhibiting histological characteristics of the mass. (A) HE 40X; proliferation of neoplastic mesenchymal cells with multifocal to coalescent mineralization; (B) HE 100X; formation of frequently mineralized trabecular osteoid matrix; (C) HE 40X; presence of three distinct morphologic groups of mesenchymal cells: fusiform, chondroid and osteoid; (D) HE 400X; neoplastic cells with nuclear atypicalness associated with countless mitotic figures.

After two weeks post-diagnosis, the patient underwent an exploratory laparotomy and complete excision of the mass. The mass exhibited moderate adhesion to adjacent tissues, necessitating the removal of a jejunal segment (Figure 3). The excised tissue was sent for histological evaluation, revealing a malignant, expansive, and partially encapsulated proliferation of mesenchymal cells. These cells exhibited fusiform, osteoid, and chondroid morphologies. The fusiform cell population displayed marked anisocytosis, anisokaryosis, and multiple mitotic figures. In contrast, the osteoid and chondroid cell populations showed moderate anisocytosis and anisokaryosis, with few mitotic figures. The mass’s central portion was characterized by extensive necrosis (Figure 4). The histopathological diagnosis confirmed the presence of a primary extraskeletal osteosarcoma in the omentum.

The patient exhibited signs of recovery post-surgery. However, 27 days later, the patient presented with weight loss. Subsequent ultrasonographic and radiographic examinations were conducted due to clinical suspicion of local recurrence or peritonitis.

**Figure 5:**
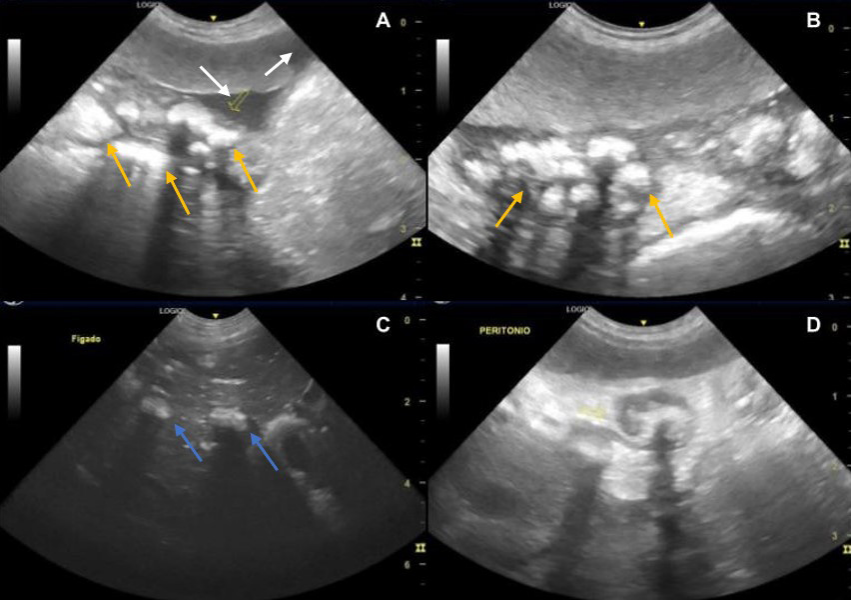
Ultrasonographic images, 27 days after surgery. (A) Image exhibiting free abdominal fluid (white arrows). (A) and (B) hyperechoic multifocal lesions scattered throughout the abdomen, with posterior acoustic shadowing (yellow arrows); (C) Liver presenting with permeated hyperechoic areas with posterior acoustic shadowing (blue arrows); (D) Increased echogenicity and heterogeneous aspect of fatty tissues.

The ultrasound results indicated local recurrence and metastasis, characterized by multifocal mineral lesions with acoustic shadowing artifacts diffusely spread across the abdomen. These affected the liver, omentum, and mesentery. Additionally, a new mass, measuring 4.50 x 3.66 cm, was identified in the same location as the previously removed one. Abdominal effusion and signs of partial intestinal obstruction were also observed, potentially associated with post-surgical adhesions (Figure 5).

**Figure 6:**
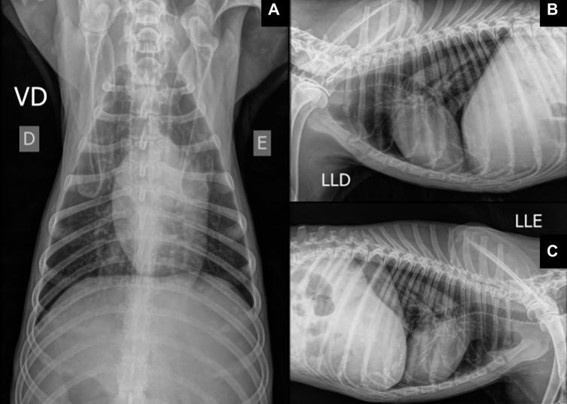
Thoracic radiographs, post-surgical staging (27 days). (A) Ventrodorsal view (VD - ventro-dorsal); (B) Right lateral view (LLD - látero-lateral direita); and (C) Left lateral view (LLE - látero-lateral esquerda), showing no evidence of metastasis.

**Figure 7:**
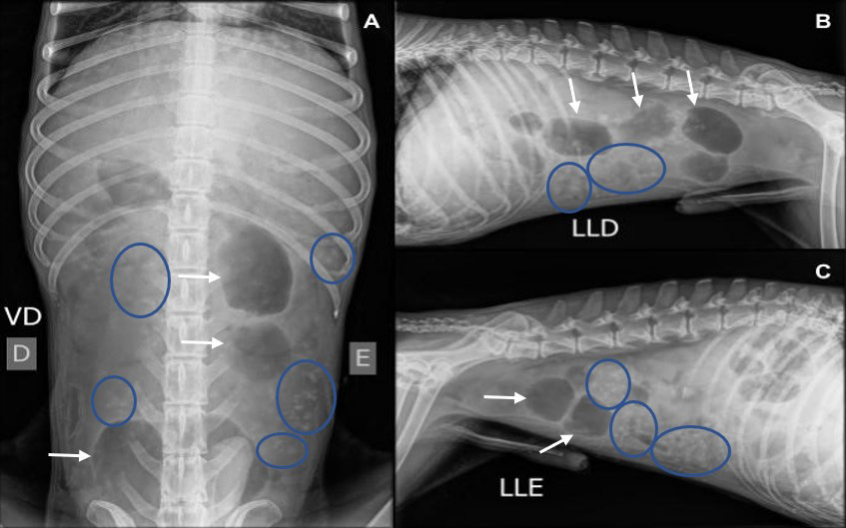
Abdominal radiographs, post-surgical staging (27 days). (A) Ventrodorsal (VD - ventro-dorsal) view with multiple small mineral lesions diffusely distributed (blue circles), as well as a large amount of gas in some intestinal segments (white arrows), compatible with the ultrasound findings; (B) Right lateral view (LLD - látero-lateral direita); and (C) left lateral view (LLE - látero-lateral esquerda), complementary to the first view.

The thoracic radiographs revealed no evidence of metastasis (Figure 6). However, multiple multifocal mineral spots were discernible in the abdominal radiographs, corroborating the findings from the ultrasound images (Figure 7).

**Figure 8:**
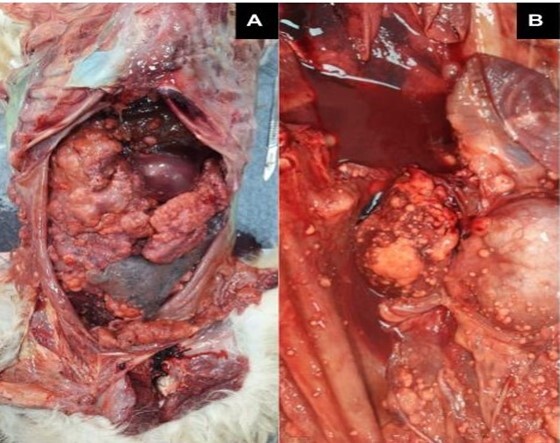
Necropsy pictures. (A) and (B) Whitened lesions (calcifications) of several dimensions, dispersed throughout the abdominal cavity and parietal peritoneum.

**Figure 9:**
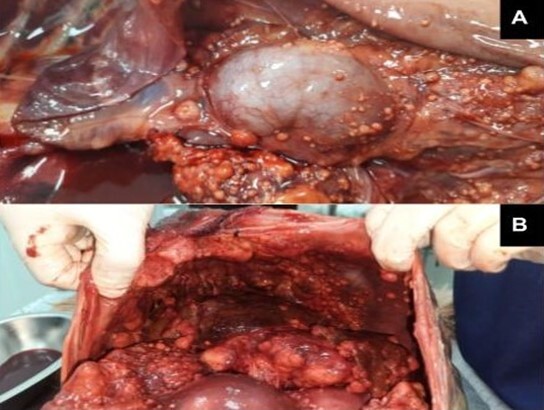
(Continued) Necropsy pictures. (A) Whitened lesions (calcifications) dispersed throughout the omentum; and (B) Similar lesions adhered to the muscle wall.

Forty-two days post-surgery, due to clinical deterioration, euthanasia was carried out. The body was subsequently sent for necropsy. The necropsy confirmed the sonographic exam findings, revealing multiple mineral lesions diffusely scattered across the omentum, adhered to the parietal peritoneum, and within the hepatic parenchyma (Figures 8 and 9).

## Discussion

The patient discussed in this study was monitored from initial diagnosis through to euthanasia and necropsy. The symptoms presented were consistent with those found in the literature, being nonspecific and primarily associated with the abdominal location of the tumor, which carried a poor prognosis (Duffy et al., 2017; Kuntz et al., 1998; Langenbach et al., 1998). The epidemiological characteristics were also in line with previous findings, such as advanced age (Kuntz et al., 1998; Langenbach et al., 1998; Thompson & Dittmer, 2017). Factors such as sex, breed, and size were found to be unrelated to the diagnosis (Langenbach et al., 1998; Leonardi, 2022; Patnaik, 1990; Thompson & Dittmer, 2017; Urbiztondo et al., 2010).

The diagnosis was confirmed through an initial ultrasound evaluation, followed by supplementary radiographs and histopathological confirmation, in accordance with the histological features described (Guim et al., 2019; Kuntz et al., 1998; MacKenzie et al., 2012; Patnaik, 1990; Piedra-Mora et al., 2016; Urbiztondo et al., 2010). The blood test results were nonspecific to the case and could be indicative of an inflammatory or neoplastic process. A clinical examination of the patient was conducted to eliminate the possibility of bone disorders that could negate the diagnosis of primary extraskeletal osteosarcoma.

The patient underwent a surgical procedure involving complete resection of the mass, as recommended (Duffy et al., 2017). However, no chemotherapy was associated with the treatment. The patient survived for 42 days post-surgery, after which euthanasia was performed due to a deteriorating clinical condition, local recurrence of the tumor, and diffuse abdominal metastasis (Langenbach et al., 1998; Thompson & Dittmer, 2017). The cause of death was not directly attributable to the tumor but to euthanasia. Consequently, the patient’s survival time was slightly longer than the average reported in veterinary literature, which is 33 days for patients who underwent surgery without chemotherapy and whose death was directly related to the neoplasm (Kuntz et al., 1998).

## Conclusion

Extraskeletal osteosarcoma is a rare and highly malignant neoplasm in dogs, characterized by its infiltrative and metastatic potential. This condition often carries a poor prognosis, particularly in cases with an abdominal presentation. The literature contains few reports of cases associated with the omentum, as in the case discussed in this study. The efficacy of available treatments remains unproven due to the challenges in diagnosing this rare condition and the lack of statistical and supportive data. Given the nonspecific nature of the symptoms, abdominal ultrasound played a crucial role in the initial diagnosis due to its accessibility. Although it lacks specificity, it is highly sensitive in detecting abdominal masses, thereby aiding in decision-making regarding case management.
